# The complete chloroplast genome and phylogenetic analysis of *Schisandra henryi* C. B. Clarke

**DOI:** 10.1080/23802359.2026.2621450

**Published:** 2026-01-29

**Authors:** Xiuying Yang, Xuefei Qiu, Xianglan Liang, Song Guo, Guoan Shen

**Affiliations:** a College of Biology and Food Engineering, Guangxi Science & Technology Normal University, Laibin, PR China; b School of Chemistry and Chemical Engineering, Guangdong Pharmaceutical University, Guangzhou, PR China; c College of Smart Agriculture (College of Internet of Things Engineering), Guangxi Science & Technology Normal University, Laibin, PR China; d Institute of Medicinal Plant Development, Chinese Academy of Medical Sciences & Peking Union Medical College, Beijing, PR China

**Keywords:** Chloroplast, *Schisandra henryi*, genome, phylogenetic analysis

## Abstract

*Schisandra henryi* C.B. Clarke belongs to the Schisandraceae family and is known to have important medicinal benefits. The complete chloroplast genome of *S. henryi* was 146,882 bp in length, with the overall GC content of 39.45%. 124 genes were annotated, which consist of 81 protein coding genes, 35 tRNA genes, and 8 rRNA genes. The phylogenetic tree constructed based on chloroplast genomes indicates that the genus *Schisandra* is closely related, with *S. henryi* being most closely related to *S. sphenanthera* within this group. This study provides further insights into the chloroplast genome database of *S. henryi.*

## Introduction

*Schisandra henryi* C.B. Clarke belongs to the Schisandraceae family and is mainly distributed in Yunnan Province, China (Jafernik et al. 2023). In traditional medicine, the roots and stems of *S. henryi* exhibit wind-damp-dispelling, blood-activating, and analgesic properties, whereas the fruits demonstrate lung-astringing, antitussive, antiperspirant, and essence-stabilizing effects (Ye et al. 2021). It contains various medicinal components, including lignans, polyphenols, triterpenoids, and nortriterpenoids, among others (Jafernik et al. 2023). Schiprolactone A, schisanlactone B, nigranoic acid, and schisandronic acid, which were first isolated from the stems of *S. henryi*, exhibit certain cytotoxic activity (Chen et al. 2003; Xue et al. 2011). *S. henryi* extracts show anti-inflammatory and antioxidant activities (Jafernik et al. 2024). Additionally, compounds such as henridilactones E, H, N, and O and schinortriterpenoids display neuroprotective effects and promote neurite outgrowth (He et al. 2020; Jafernik et al. 2024). Studies have shown that extracts from *in vitro*-cultured microshoots and leaves of parent plants of *S. henryi* demonstrated significant antiproliferative activity against human cancer cell lines (including Jurkat, MCF-7, HT-29, and HeLa), along with certain antimicrobial activities (Jafernik et al. 2024).

While the medicinal properties of *S. henryi* have been well studied, research on its genetic and genomic aspects remains scarce. Schisandraceae is a small family of basal angiosperms within the order Austrobaileyales, comprising several genera including *Schisandra*. The chloroplast (cp) genome, as a highly conserved and informative DNA molecule, provides valuable information for phylogenetic and evolutionary studies and can serve as a ‘super-barcode’ for species identification and germplasm authentication (Wang et al. 2018; Milarska et al. 2023).

## Materials and methods

**Figure 1.**
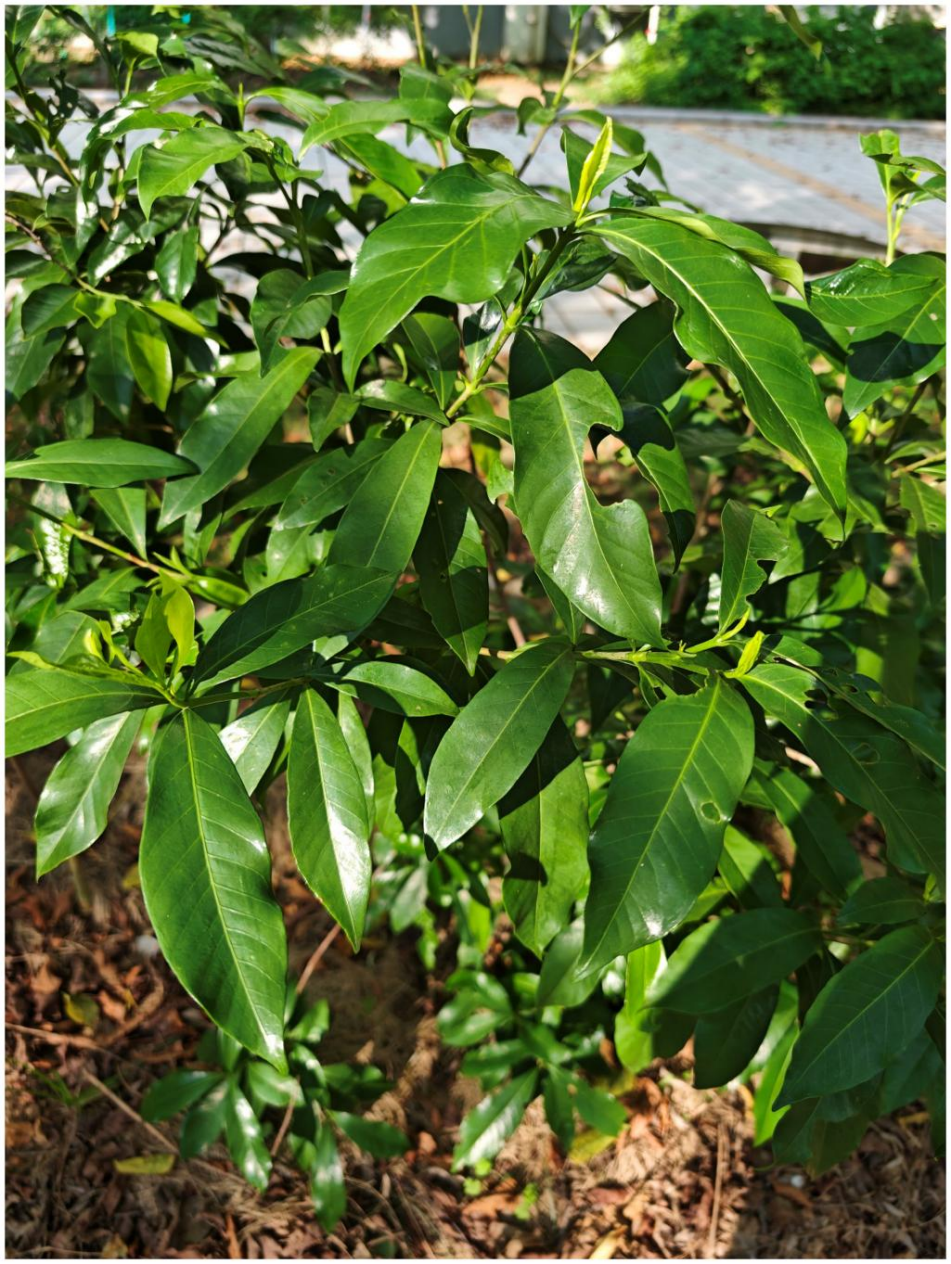
Plant image of *S. henryi. S. henryi* is a climbing vine with thin, glossy, elliptic-ovate leaves with slightly serrated margins, alternate inflorescences, and a reticulate vein structure. The photo of the species was taken by the authors (Song Guo).

Fresh leaves of one individual were collected from Rongshui Miao Autonomous County, Liuzhou City, Guangxi Zhuang Autonomous Region, China (N23°47′07″, E109°11′46″) and preserved in desiccant. The collection of plant materials (Figure 1) was authorized by Dr. Song Guo (guosong0804@163.com). A voucher specimen of *S. henryi* (voucher number: JXHC031) is preserved at the College of Biology and Food Engineering, Guangxi Science and Technology Normal University, under the supervision of Dr. Song Guo. Species identification was performed by Dr. Song Guo based on morphological characteristics in accordance with *Flora of China*.

Total genomic DNA was extracted from fresh leaf tissue using the DNeasy Plant Mini Kit (Cat. No. 69104, Qiagen, Hilden, Germany). A DNA library was then created using insert fragments of approximately 300 base pairs in size, followed by paired-end sequencing (2 × 150 bp) on the Illumina HiSeq 2500 platform (San Diego, CA). The quality control of the raw sequencing reads was performed using the fastp v0.2 (Chen et al. 2018), resulting in the production of high-quality filtered reads. Subsequently, the reads were utilized for the *de novo* assembly of complete plastid genomes, employing the GetOrganelle v1.7.7.1 (Jin et al. 2020). The assembly graph was visualized using Bandage v0.8.1 (Wick et al. 2015). The assembled cp genome was annotated using CPGAVAS2 (Shi et al. 2019). The annotated genome was visualized using CPGView (Liu et al. 2023). Subsequently, cis-splicing and trans-splicing genes were also identified and analyzed based on the annotation results and visualized using the CPGView.

A total of 15 cp genome sequences of species from the family Schisandraceae were retrieved from the NCBI database for phylogenetic analysis, with *Astelia australiana* NC_045865.1 (Asteliaceae) used as the outgroup. Sequence alignment and phylogenetic tree construction were performed using PhyloSuite v1.2.3 (Zhang et al. 2020). The multiple sequence alignment was conducted with MAFFT (Katoh et al. 2002). A maximum-likelihood phylogenetic tree was then inferred using IQ-TREE (Nguyen et al. 2015) under the GTR + F + I + G4 substitution model. Branch support was assessed with 1000 replicates of ultrafast bootstrap (UF-bootstrap) and the SH-like approximate likelihood ratio test (SH-aLRT) with 1000 replicates. The resulting tree was visualized using the iTOL (Letunic and Bork 2021) web platform.

## Results

**Figure 2.**
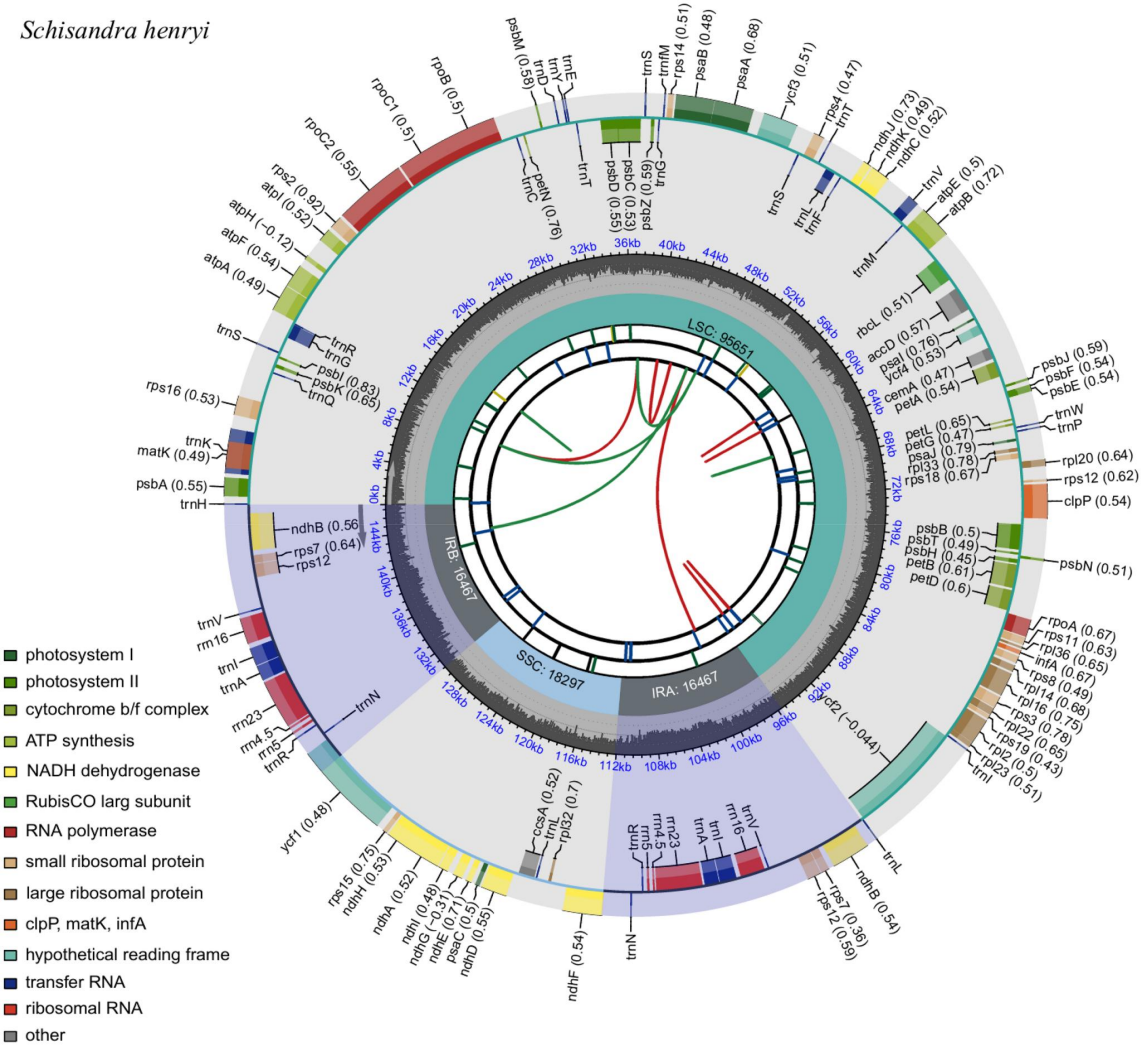
Circular visualization of the plastome architecture. The schematic comprises six concentric tracks: (1) innermost repeat elements (direct/palindromic repeats shown as red/green arcs); (2) long tandem repeats (blue bars); (3) microsatellites (color-coded by unit size: p1–p6); (4) structural boundaries (SSC/IR/LSC regions); (5) GC content profile; and (6) outermost gene features (color-coded by function, with transcriptional orientation indicated). Codon usage bias appears parenthetically after gene names. Species identification is noted in the upper left, with functional legend in the lower left.

Approximately, 4.1 Gb of raw data of *S. henryi* was obtained. The cp genome exhibited a length of 146,882 bp. The coverage depth ranged from a minimum of 115× to a maximum of 24,373× (Figure S1). All gene features, such as cis-splicing (Figure S2) and trans-splicing (Figure S3), as well as circular map (Figure 2), were visualized using CPGView. The cp genome had a typical quadripartite structure, consisting of a large single-copy (95,651 bp), a small single-copy (18,297 bp), and two inverted repeat (IR) regions (16,467 bp). The average guanine–cytosine (GC) content was 39.45%.

A total of 124 functional genes were annotated, among which 81 are protein-coding genes (PCGs), 35 were transfer genes (tRNAs), and eight are ribosomal RNA genes (rRNAs). Within the IR regions, there are duplications of three PCGs (*ndhB*, *rps*12, *rps*7), five tRNAs (*trnN-GUU*, *trnR-ACG*, *trnA-UGC*, *trnI-GAU*, *trnV-GAC*), and four rRNAs (*rrn*5, *rrn*4.5, *rrn*23, and *rrn*16). Furthermore, 10 PCGs contained a single intron (*rps*16, *atpF*, *rpoC*1, *petB*, *petD*, *rpl*16, *rpl*2, *ndhB*(2x), *ndhA*), and two genes (*ycf*3 *and clpP*) contained two introns (Figure S2). Meanwhile, eight tRNAs contained single intron (*trnK-UUU*, *trnG-GCC*, *trnL-UAA*, *trnV-UAC*, *trnI-GAU*(2x), and *trnA-UGC* (2x)). The cp genome’s trans-splicing gene *rps*12 (Figure S3) comprised three unique exons, two of which were duplicated due to their localization in the IR regions.

**Figure 3.**
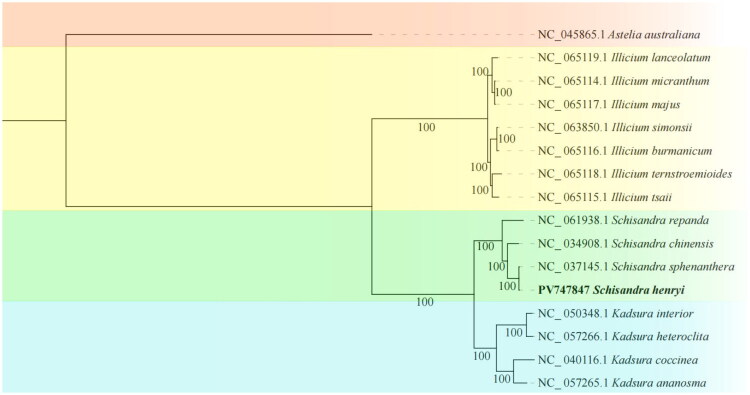
Systematic evolutionary tree of *S. henryi*. Download 15 chloroplast genome sequences from NCBI and construct a phylogenetic tree using the maximum-likelihood method. The Schisandraceae species are *Illicium lanceolatum* NC_065119.1, *Illicium micranthum* NC_065114.1, *Illicium majus* NC_065117.1, *Illicium simonsii* NC_063850.1 (Tiantian et al. 2025), *Illicium burmanicum* NC_065116.1, *Illicium ternstroemioides* NC_065118.1, *Illicium tsaii* NC_065115.1, *Schisandra repanda* NC_061938.1, *Schisandra sphenanthera* NC_037145.1, *Kadsura interior* NC_050348.1, *Kadsura heteroclita* NC_057266.1, *Kadsura ananosma* NC_057265.1 (Liu et al. 2020), *Kadsura coccinea* NC_040116.1 (Li and Zheng 2018), *Schisandra chinensis* NC_034908.1, with *Astelia australiana* NC_045865.1 (Amor et al. 2020) as the outgroup.

The ML phylogenetic tree constructed from the Schisandraceae family showed that *S. henryi* and *Schisandra sphenanthera* were most closely related (Figure 3). The family Schisandraceae comprises only three genera, among which *Schisandra* and *Kadsura* are more closely related to each other than to *Illicium* (Figure 3).

## Discussion and conclusions

The complete cp genome of *S. henryi* exhibited a typical circular quadripartite structure and had a complete size of 146,882 bp, containing a total of 124 genes, with a high average sequencing depth of 397.90×, which ensured high-quality assembly and subsequent analysis. The cp genomes of *Schisandra* species (Li and Zheng 2018; Wei et al. 2020; Chen et al. 2024; Zhang et al. 2024; Yu et al. 2025). Phylogenetic analysis shows that species within the genus cluster together with high support (bootstrap values of 100%), with *S. henryi* being most closely related to *S. sphenanthera*. The cp genome of *S. sphenanthera* (accession number: NC_037145.1) was reported to be 146,853 bp in length (Wei et al. 2020), which was 29 bp smaller than the cp length of *S. henryi.* The sequence variation among *Schisandra* species allows differentiation between species, while the conserved structure and gene content of the cp genome make it a reliable ‘super-barcode’ for species identification and germplasm authentication.

Overall, the complete cp genome of *S. henryi* enriches the genomic resources available for the genus and provides valuable data for future comparative, phylogenetic, and evolutionary studies in Schisandraceae.

## Supplementary Material

Supplemental Material

## Data Availability

The genome sequence data that support the findings of this research are available in GenBank under the accession number PV747847. The BioProject, BioSample, and SRA numbers are PRJNA1271945, SAMN48890229, SRR33835836, respectively.
