# Supplementary material for: The complete chloroplast genome and phylogenetic analysis of Schisandra henryi C. B. Clarke
Source: Mitochondrial DNA B Resour. 2026 Jan 29;11(3):335–9. doi: 10.1080/23802359.2026.2621450 (PMC12857699; doi:10.1080/23802359.2026.2621450)
Supplement: Supplemental Material [file TMDN_A_2621450_SM4430.docx]

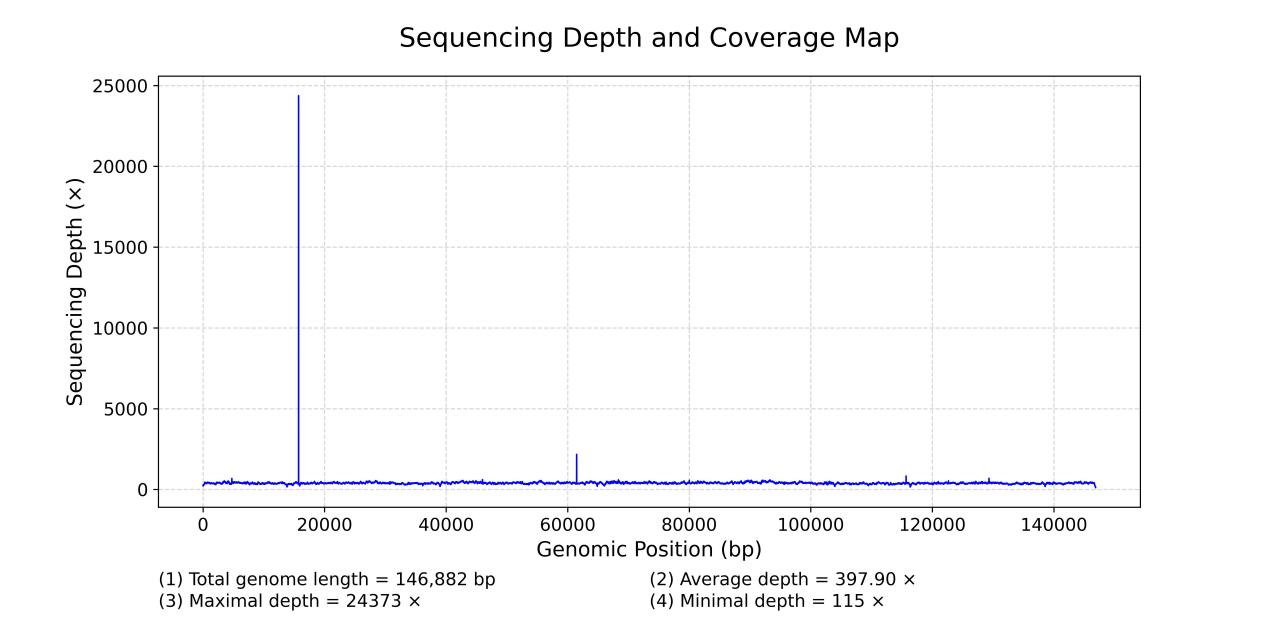
**Figure S1.** Displays the sequencing depth profile of *S. henryi* chloroplast genome (146,882 bp). The depth distribution shows: Peaks in IR regions (max 24,373×) due to duplicated rRNA operons, Valleys in SSC (min 115×) potentially caused by GC bias, Genome-wide average depth of 397.90× ensures high-confidence variant calling. The bimodal pattern is characteristic of angiosperm chloroplasts with quadripartite structure.


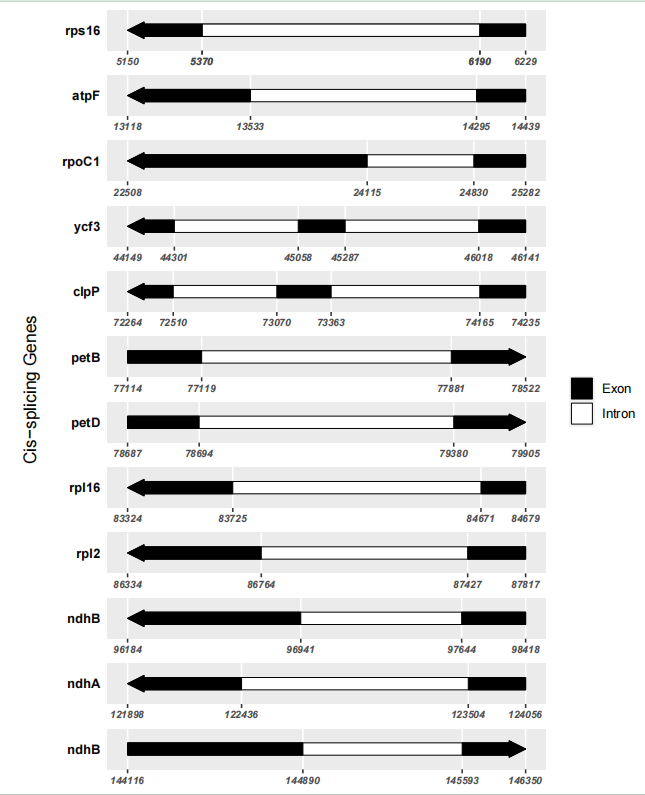


**Figure S2.** Gene organization of cis-spliced loci in the *S. henryi* plastome. Arrows denote transcriptional direction, with exons (black) and introns (white) depicted. Gene names are listed on the left, and genomic positions are annotated below.


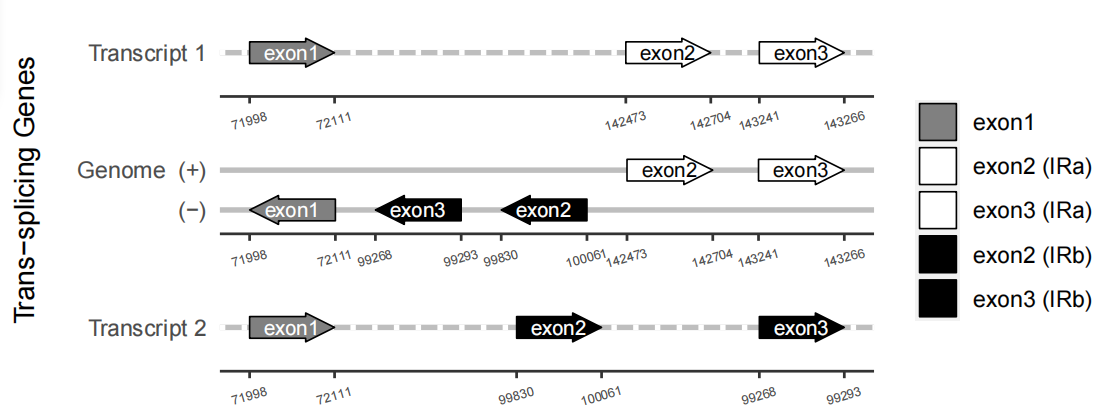


**Figure S3.** Mapping of the bipartite *rps12* gene exhibiting trans-splicing in the *S. henryi* chloroplast genome.

**Table S1**. Comparative features of the published chloroplast genomes of *Schisandra* species. The table lists the GenBank accession numbers, genome lengths, GC content, and the numbers of total genes, protein-coding sequences (CDS), and RNA genes for each species.

| Species | Genbank | Length (bp) | GC (%) | Total Genes | CDS | tRNA | rRNA |
| --- | --- | --- | --- | --- | --- | --- | --- |
| *S. henryi* | PV747847.1 | 146,882 | 39.45 | 124 | 81 | 35 | 8 |
| *S.chinensis* | KU362793.1 | 147,772 | 39.48 | 125 | 82 | 35 | 8 |
| *S. sphenanthera* | MK193856.1 | 146,853 | 39.59 | 125 | 83 | 34 | 8 |
| *S. sphenanthera* | NC_037145.1 | 146,843 | 39.60 | 125 | 82 | 35 | 8 |
| *S. propinqua* | PQ037862.1 | 145,302 | 39.69 | 124 | 82 | 34 | 8 |
| *S. repanda* | NC_061938.1 | 146,620 | 39.66 | 125 | 82 | 35 | 8 |
| *S. macrocarpa* | PP566608.1 | 144,288 | 39.75 | 125 | 82 | 35 | 8 |
| *S. plena* | PQ282136.1 | 145,490 | 39.68 | 126 | 83 | 35 | 8 |
